# Antioxidant Activity, Inhibition of Intestinal Cancer Cell Growth and Polyphenolic Compounds of the Seagrass *Posidonia oceanica*’s Extracts from Living Plants and Beach Casts

**DOI:** 10.3390/md22030130

**Published:** 2024-03-11

**Authors:** Alkistis Kevrekidou, Andreana N. Assimopoulou, Varvara Trachana, Dimitrios Stagos, Paraskevi Malea

**Affiliations:** 1 Laboratory of Organic Chemistry, School of Chemical Engineering, Aristotle University of Thessaloniki, 54124 Thessaloniki, Greece; alkistiskebrek@gmail.comadreana@gapps.auth.gr (A.N.A.); 2 Environmental Engineering Laboratory, Department of Chemical Engineering, Aristotle University of Thessaloniki, 54124 Thessaloniki, Greece; 3 Department of Biology, Faculty of Medicine, School of Health Sciences, University of Thessaly, Biopolis, 41500 Larissa, Greece; vtrachana@med.uth.gr; 4 Department of Biochemistry and Biotechnology, School of Health Sciences, University of Thessaly, Biopolis, 41500 Larissa, Greece; 5 Department of Botany, School of Biology, Aristotle University of Thessaloniki, 54124 Thessaloniki, Greece

**Keywords:** *Posidonia oceanica*, living marine angiosperm, dead mass of angiosperm, antioxidant capacity, antiproliferative activity, free radicals, polyphenolic compounds

## Abstract

The aim of the present study was to investigate the use of *Posidonia oceanica* for making products beneficial for human health. Firstly, we demonstrated that the antioxidant defense (i.e., SOD and APX activity) of *P. oceanica*’s living leaves (LP) has low efficacy, as they partly neutralize the produced H_2_O_2_. However, high H_2_O_2_ levels led LP to produce, as a response to oxidative stress, high phenolic content, including chicoric acid, *p*-coumaric acid, caftaric acid, *trans*-cinnamic and rutin hydrate, as shown by UHPLC-DAD analysis. In addition, LP extracts inhibited intestinal cancer cell proliferation. Moreover, *P. oceanica’s* beach casts consisting of either Wet ‘Necromass’ (WNP) or Dry ‘Necromass’ (DNP) were used for preparing extracts. Both DNP and WNP exhibited antioxidant and antiproliferative activities, although lower as compared to those of LP extracts. Although both *P. oceanica’s* meadows and beach casts are considered priority habitats in the Mediterranean Sea due to their high ecological value, legislation framework for beach casts forbidding their removal is still missing. Our results suggested that both LP and DNP could be utilized for the production of high-added value products promoting human health, provided that a sustainability management strategy would be applied for *P. oceanica’s* meadows and beach casts.

## 1. Introduction

The ecological value of the most abundant, endemic, long-lived seagrass species *Posidonia oceanica* (Linnaeus) Delile in the coastal systems of the Mediterranean Sea is very important [1,2,3,4,5]. Despite the fact that its meadows are considered priority habitats (1120*, Directive 92/43/EEC, NATURA 2000 Network), they remain among the most vulnerable aquatic systems (United Nations Environment Programme, UNEP 2020) and are protected by international conventions [6,7]. Given that the beach casts and ‘*banquettes*’ formed by the dead mass of the fallen *P. oceanica* leaves washing up on shores along with broken rhizomes, protect the coasts from strong waves, support important food webs and are a source of nitrogen and organic carbon (e.g., POSBEMED 2 2021) [8], they are considered to be priority habitats. However, in Mediterranean countries, specific legislation at the basic scale forbidding *P. oceanica* beach cast removal is still missing and each country adopts its existing national regulations. Thus, different management strategies apply, depending on different socio-economic contexts [9]. *P. oceanica* beach casts often are treated as important resources and sometimes as waste, with the municipal authorities taking the initiative to remove them from the coasts and dumped in landfills, without any processing. Taking into account the interest of the beach casts/‘*banquettes*’ ecosystem, and the possibility of their utilization as a resource of products having beneficial effects on human health, management programs have been proposed, such as their partial removal from the shores (e.g., 20% of the mass, after separating any sediment) [8,9].

Since 70% of the earth’s surface is covered by seas and oceans, marine organisms can be an important source of new bioactive compounds promoting human health [10]. Seagrasses have various uses for humans, such as food, medicine, and biofuels, while several of their compounds have exhibited antioxidant, anticancer, anti-inflammatory, antibacterial, antiviral, antidiabetic, vasoprotective and hemolytic activities [10,11,12]. Recent review articles [10,11] summarized the bioactive components isolated from marine seagrasses such as *Posidonia oceanica*, *Thalassia ciliatum*, *T. hemprichii*, *T. testudinum*, *Zostera marina*, *Z. noltii*, *Cymodocea nodosa, C. serrulata, Enhalus acoroides, Ruppia maritima, R. cirrhosa, Halophila stipulacea* and *H. ovalis.* However, the activities of bioactive substances derived from marine macrophytes, especially that of the seagrasses, have not been thoroughly investigated, due to their great biodiversity [13]. Recently, research interest has turned towards the characterization of the *P. oceanica* bioactive components [14,15,16,17,18,19]. Additionally, *P. oceanica* leaf dead mass is used for the manufacture of glassware, roof insulation, animal feed, fertilizers, energy production [14,15,18] and even the detection of new bioactive compounds after cultivation of their associated microbial community [19].

As seagrasses grow worldwide, in a variety of coastal and estuarine environments, they are expected to be exposed to a multitude of abiotic and biotic environmental stresses [2,3,20,21,22,23]. These stressors have been shown to result in an increase in the production of Reactive Oxygen Species (ROS), such as hydroperoxyl (HO_2_^•^), superoxide (O_2_^•−^), hydroxyl (OH^•^), alkyl peroxyl (RO_2_^•^) and alkoxyl (RO^•^) radicals, singlet oxygen (^1^O_2_*) and hydrogen peroxide (H_2_O_2_) [24,25,26,27]. Extreme amounts of ROS lead to oxidative damage in plant biomolecules (e.g., proteins, lipids, nucleic acids) [28]. Thus, oxidative stress stimulates enzymatic (i.e., SOD, CAT, APX) [24,25,26,27,29,30] and non-enzymatic antioxidant mechanisms [29,30] in seagrasses. The antioxidant activity has been studied in the living leaves of different seagrass species such as *Posidonia oceanica*, *Zostera marina*, *Thalassia testudinum*, *T. hemprichii*, *Thalassodendron ciliatum*, *Halodule pinifolia* and *Spyringodium filiforme* [31,32,33,34,35,36,37,38,39,40]. However, the relevant studies are too limited for the seagrass’ dead mass [16,17,41]. Extracts from seagrasses have displayed a high ability to neutralize free radicals, as they are a rich source of secondary metabolites, such as the phenolic compounds *p*-coumaric, caffeic, gallic, chicoric, *trans*-ferulic, caftaric, protocatechuic, *p-*hydroxybenzoic, vanillic acid, gentisic acid and quercetin [42,43,44,45,46]. Specifically, 51 polyphenolic compounds have been identified in the living plants of *P. oceanica* (reviewed in [47]). Additionally, extracts of living plants of *Posidonia oceanica* have been found to exhibit antiproliferative and antimetastatic effects against various cancer cells, such as colon, breast, cervical, prostate and neuroblastoma [48,49].

Since, as mentioned above, there have been so far only a few studies on the *P. oceanica*’s dead mass, the present study aimed to investigate (a) the living leaves (LP), (b) the dead leaves deposited on the coast at sea level where the waves break (WNP) and (c) the beach casts/‘*banquettes*’ (DNP) of the seagrass *P. oceanica* (collected from the Epanomi coast, the Thermaikos Gulf, the Aegean Sea, Greece) the production of ROS (i.e., H_2_O_2_) and the activities of the antioxidant enzymes SOD and APX, and the protein content. Moreover, we aimed to analyze the free radical scavenging and reducing power activities, as well as the inhibition of proliferation against human colon cancer cells of the extracts prepared from LP, WNP and DNP. Moreover, the identification and quantification of the *P. oceanica* extracts’ phenolic compounds were made, using UHPLC-DAD. Finally, the broader collection area of *P. oceanica* was used as a ‘case study’ in order to estimate the extracts’ phenolic amount that could be produced, based on the DNP’s mass, or could be used, taking into consideration the management strategies. This estimation could contribute to management plans for utilizing part of the produced *P. oceanica*’s leaf biomass and/or its dead mass for creating high-added value products for human health, while having the least impact on the ecosystems. Currently, there is no legislation framework in Mediterranean countries for the removal and management of *P. oceanica*’s *‘banquettes’*, and therefore such evaluations are of great importance.

## 2. Results

### 2.1. Phenological Parameters of Posidonia oceanica Plants, and Its Meadow and ‘Banquette’ Characteristics

**Figure 1:**
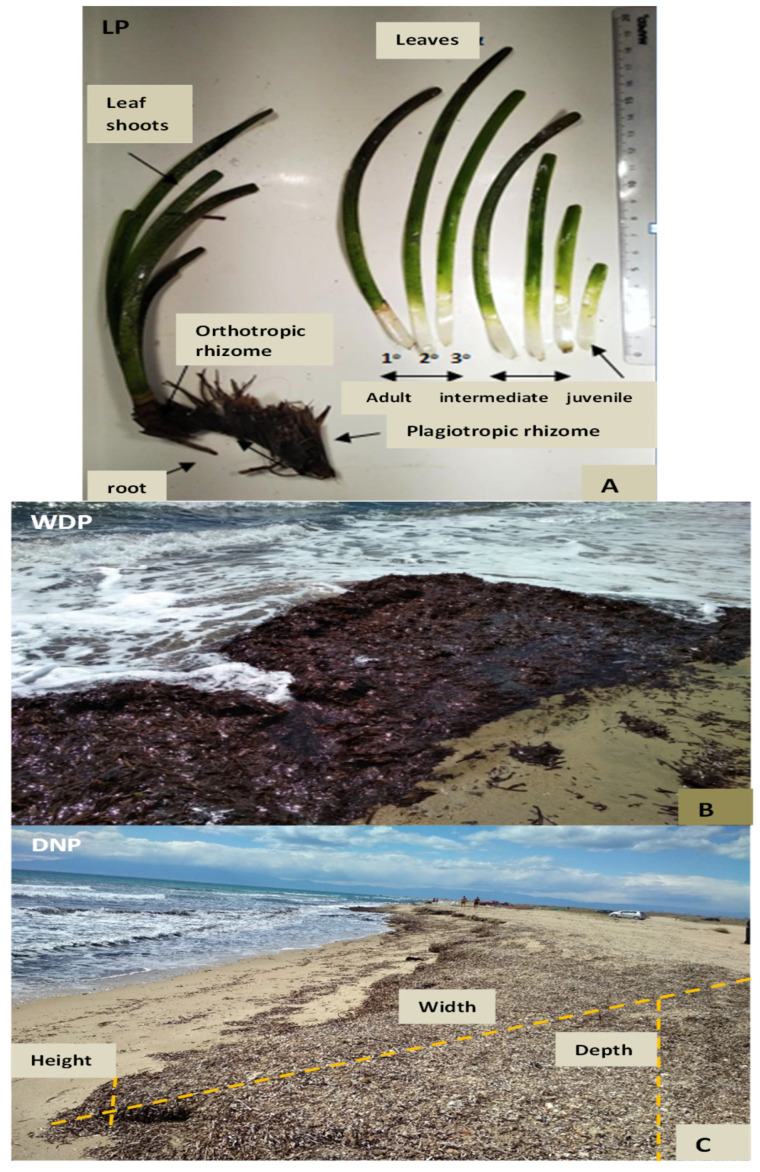
(**A**) Macroscopic view of *P. oceanica* living plants (LP) and division into plant parts, (**B**) Wet ‘Necromass’ (WNP) of *P. oceanica* leaves, deposited on the shores at the sea level, and (**C**) Dry ‘Necromass’ (DNP) of *P. oceanica,* and illustration of the width, depth and height of the DNP in Potamos station, Epanomi coast, Thermaikos Gulf (Aegean Sea).

The phenological parameters (mean ± SE) of *P. oceanica* at the individual and population level were the following: the total number of leaves/shoot^−1^—4.181 ± 0.037, n = 347; the number of the adult leaves shoot^−1^—2.386 ± 0.051, n = 193; the intermediate leaves shoot^−1^—2.410 ± 0.043, n = 200; the juvenile leaves shoot^−1^—1.062 ± 0.029, n = 69; and the leaf surface area (cm^2^ shoot^−1^) of the adult and intermediate leaves (Figure 1A), based on their leaf length and width—38.57 ± 1.54, and 68.45 ± 3.92, n = 25, respectively.

The range of the weight loss (%) of the living leaves (LP) was 78.3–85.8%, and it was higher in the adult leaves and lower in the juvenile leaves, whereas the respective values of the Wet ‘Necromass’ (i.e., WNP) was 62.2% and of the Dry ‘Necromass’ (i.e., DNP) was 11.1% (Figure 1B,C). 

### 2.2. Oxidative Stress—Antioxidant Enzyme Production and Protein Content of Posidonia oceanica Leaves

**Figure 2:**
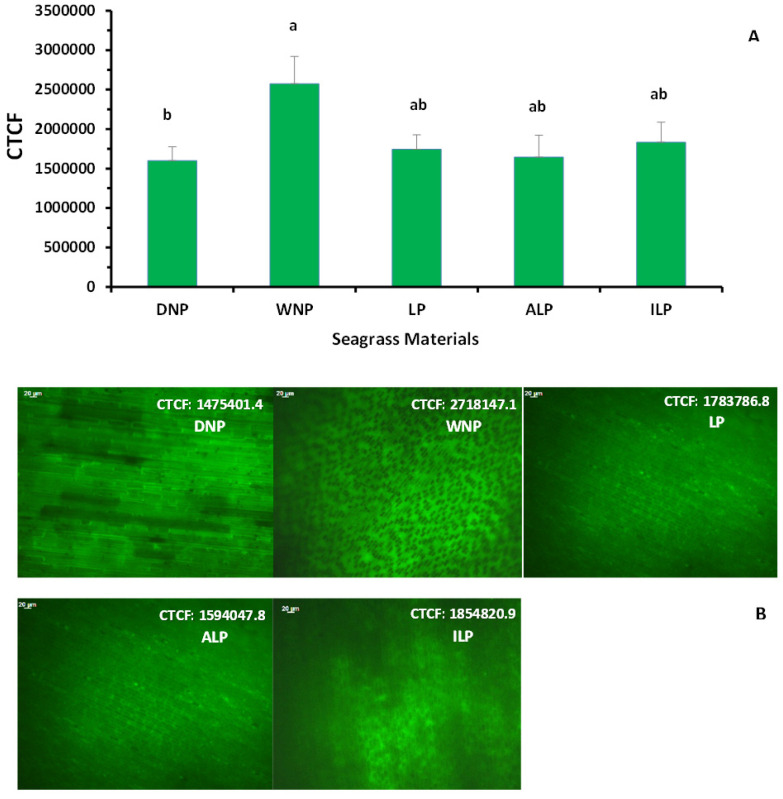
(**A**) Level of intracellular H_2_O_2_ (mean value ± standard error) in living *Posidonia oceanica* leaves (LP), separately in adult (ALP) and intermediate leaves (ILP), as well as in the Dry ‘Necromass’ (DΝP) and Wet ‘Necromass’ (WNP). The intracellular H_2_O_2_ level is expressed as the ‘Corrected Total Cell Fluorescence’ (CTCF) value of the seagrass cells. Mean CTCF values were derived from approximately 162 measurements (3 regions of 3 segments (apex, middle, base) per leaf; n = 18 leaves) and 27 measurements for ‘Necromass’ materials (3 regions per leaf piece; n = 9 leaf pieces), (**B**) hydrogen peroxide (H_2_O_2_) fluorescence images by optical microscope after staining with DCF- DA, in living leaves (LP, ALP, ILP) and in ‘Necromass’ (DNP and WNP). Scale bar: 20 µm. Different lowercase letters denote significantly different values between different plant categories (*p* < 0.05).

The levels of the intracellular hydrogen peroxide (H_2_O_2_) (mean ± SE) of various *P. oceanica* samples, expressed as the value of Corrected Total Cell Fluorescence (CTCF), as well as the H_2_O_2_ fluorescence images, obtained by fluorescence microscope, are shown in Figure 2. The CTCF values did not differ (*p* > 0.05) between the leaves of different ages [mean ± SE: 1,643,854.6 ± 277,671.5 for adult (ALP) and 1,833,417.3 ± 255,374.9 for intermediate leaves (ILP)]. Additionally, the CTCF values in the DNP samples (1,601,811.4 ± 174,439.9) were similar (*p* > 0.05) or slightly lower than those in the LP leaves (1,744,211.4 ± 183,557.8) and significantly lower (*p* < 0.05) than that in the WNP (2,572,580.5 ± 347,379.3) (Figure 2).

**Figure 3:**
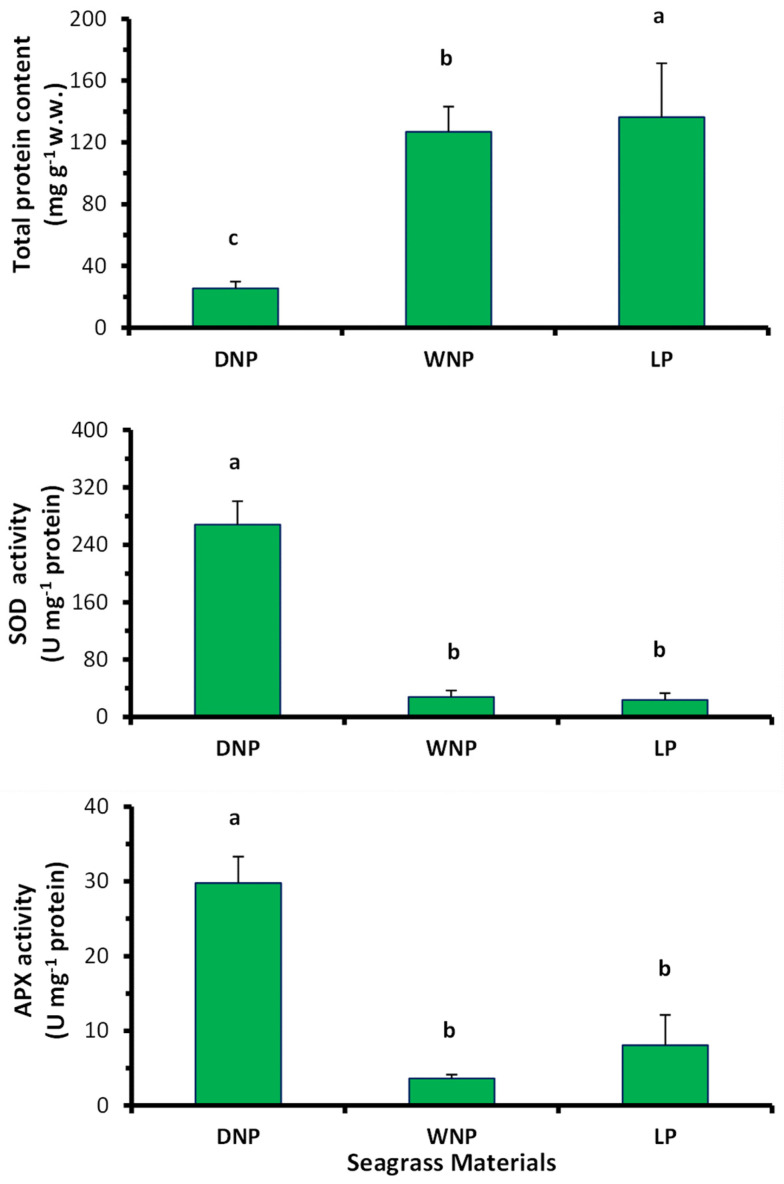
Total protein content (mg g^−1^ w.w.), activity of the antioxidant enzymes superoxide dismutase (SOD) and ascorbic peroxidase (APX) in the living leaves (LP), Wet ‘Necromass’ (WNP) and Dry ‘Necromass’ (DNP) of *P. oceanica* (mean value ± SE of three samples in each plant category and of three subsamples in each sample). Different lowercase letters denote significantly different values between different plant categories (*p* < 0.05).

As for the enzymatic antioxidant defense of *P. oceanica* materials, the mean values of SOD and APX activity of LP leaves were similar (*p* > 0.05) to those of the WNP, but significantly (*p* < 0.05) lower than that of the DNP (Figure 3). In contrast, the mean total protein content in LP leaves was significantly higher (*p* < 0.05) than that in the WNP and DNP samples (Figure 3).

The RCD % values of the analytical methods applied, from three replicates of the same extract, ranged in all seagrass materials between 0.57–19.17%, 19.18–63.14% and 17.40–52.79% for APX and SOD activity and protein content, respectively. Moreover, they were lower in LP leaves and higher in the DNP material.

### 2.3. Posidonia oceanica’s Extraction Yield

The percentage (%) yield of the extracts, calculated from the dry weight mass of the extracts, was 24% for the LP leaves, and 9% and 11% for the WNP and DNP materials, respectively.

### 2.4. Free Radical Scavenging Activity of Posidonia oceanica’s Extracts

**Figure 4:**
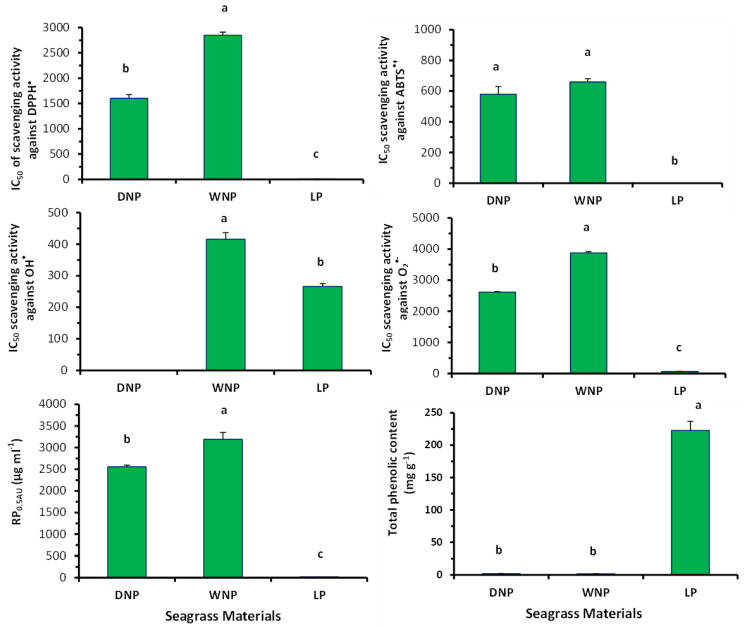
IC_50_ values (μg/mL) of scavenging activity against DPPH^•^, ABTS^•+^, OH^•^ and O_2_^•−^ radicals, RP_0.05AU_ values of reducing power, and total polyphenolic content (TPC) (mean ± SE of at least three separate experiments) of the *P. oceanica extracts* from living leaves (LP), Wet ‘Necromass’ (WNP) and Dry ‘Necromass’ (DNP) extracts. DNP extract did not exhibit IC_50_ value at the tested concentrations in the OH^•^ assay. Different lowercase letters denote significantly different values between different plant categories (*p* < 0.05).

The extracts from LP, WNP and DNP samples decreased the absorbance of the DPPH^•^ solution, at 517 nm, in a dose-dependent manner. The comparison of the IC_50_ values demonstrated that the IC_50_ value (mean ± SE: 8.2 ± 0.8 μg/mL) of LP extract was significantly lower (*p* < 0.05) than the values of the WNP and DNP extracts (2850.0 ± 60.1 and 1600 ± 75.1 μg/mL, respectively) (Figure 4 and Figure S1). This result indicated that the LP extract had a higher ability to neutralize the DPPH^•^ radical than both dead mass extracts. Additionally, the DNP extract had a higher antioxidant ability to neutralize the DPPH^•^ radical than the WNP extract (Figure 4).

The *P. oceanica* extracts decreased the absorbance of the ABTS^•+^ solution, at 730 nm, in a dose-dependent manner, indicating a significant ability to scavenge ABTS^•+^ radical. The IC_50_ value (1.6 ± 0.9 μg/mL) of the LP extract was significantly lower (*p* < 0.05) than those of WNP and DNP, whereas WNP and DNP extracts showed similar values (660 ± 20.5 and 580 ± 49.3 μg/mL, respectively) (Figure 4 and Figure S1).

Moreover, LP extract had a significantly stronger capacity (IC_50_: 267 μg/mL) to scavenge the OH^•^ radical than WNP (IC_50_: 416 μg/mL). DNP extract did not exhibit IC_50_ value at the tested concentrations (Figure 4 and Figure S2).

Likewise, LP extract had significantly (*p* < 0.05) higher scavenging capacity (IC_50_: 71.0 ± 2.0 μg/mL) against O_2_^•−^ radical than WNP (IC_50_: 3880.0 ± 36.1 μg/mL) and DNP (IC_50_: 2615.0 ± 20.2 μg/mL) extracts (Figure 4 and Figure S1). Additionally, DNP had a higher ability to neutralize O_2_^•−^ radical than WNP (Figure 4 and Figure S1).

In addition, LP extract exhibited the strongest RP activity (Figure 4 and Figure S2). Specifically, the RP_0.5AU_ values of the LP, WNP and DNP extracts were 19.5 ± 0.6, 3190 ± 75.0 and 2556 ± 57.0 mg/mL, respectively (Figure 4 and Figure S2).

The IC_50_ values of the positive control, the ascorbic acid, in all the above assays are shown in Table S1.

### 2.5. Phenolic Content of the Posidonia oceanica’s Extracts

The total phenolic content (TPC) of the LP extract (mean ± SE: 222.80 ± 13.99 mg GAE g^−1^ d.w.) was much higher (*p* < 0.05) than that of the WNP (2.12 ± 0.29 mg GAE g^−1^ d.w.) and DNP extracts (1.85 ± 0.20 GAE g^−1^ d.w.), while the WNP and DNP extracts had similar TPC (Figure 4).

**Figure 5:**
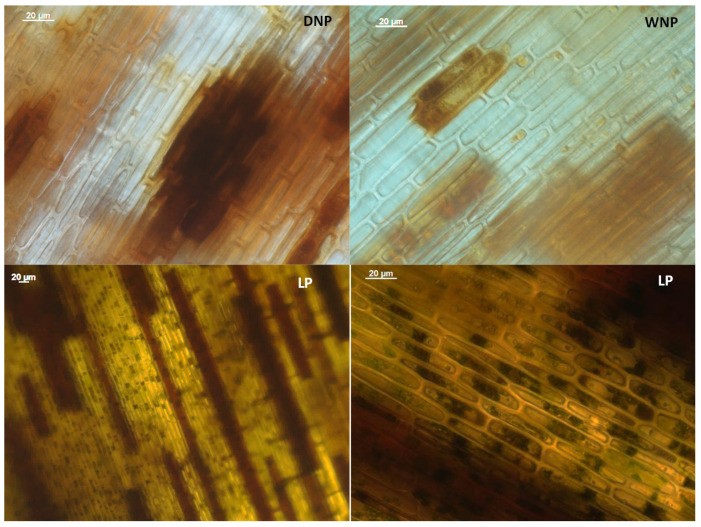
View of cells, under an optical microscope, after staining with potassium dichromate solution for estimating the content of phenolic compounds (shown as brown-orange color), in the adult living leaves (LP) of *Posidonia oceanica* (magnification 10 × 10 and 10 × 63), and in Dry ‘Necromass’ (DNP) and Wet ‘Necromass’ (WNP) (magnification 10 × 63). Scale bar: 20 µm.

Additionally, the possible presence of phenolic compounds inside the cells (dyed brown) was evident in all materials (LP, WNP and DNP). Although phenolic content seems to be higher in LP leaf cells, it is more distinct in ‘dead mass’ materials, maybe due to the disorganization of the cellular organelles (empty cells), owing to desiccation (Figure 5).

**Figure 6:**
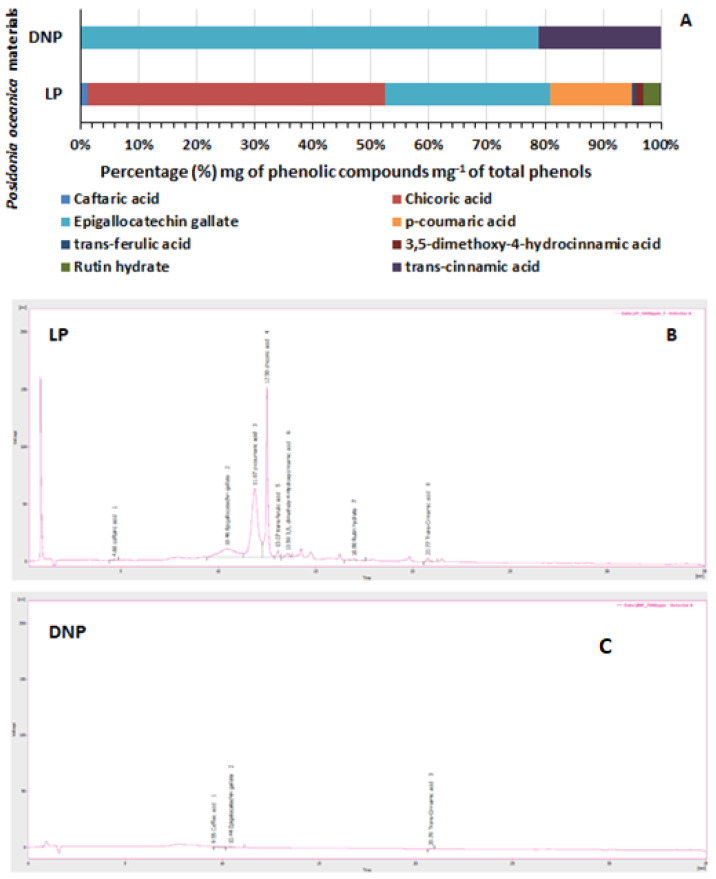
(**A**) The percentage (%) of the amount of each phenolic compound per total amount of phenols for *Posidonia oceanica*’s living leaves (LP) and the Dry ‘Necromass’ (DNP) extracts. Chromatograms of the phenolic compounds of (**B**) LP extract and (**C**) of the Dry ‘Necromass’ (DNP) extract.

Quantitative and qualitative identification of individual phenolic compounds, using HPLC analysis, showed that *P. oceanica*’s LP extracts contained the following phenolic compounds: chicoric acid, (-)-epigallocatechin gallate, *p*-coumaric acid, caftaric acid, *trans*-4-hydroxy-3-methoxycinnamic acid, rutin hydrate, *trans*-cinnamic acid and 3,5-dimethoxy-4-hydroxycinnamic acid (Table 1, Figure 6A,B). Specifically, the LP extract contained a high percentage of chicoric acid (7.059%), (-)-epigallocatechin gallate (3.933%), *p*-coumaric acid (1.916%) and rutin hydrate (0.391%) per amount of sample (Table 1). The remaining compounds were determined in smaller amounts (Table 1).

In the DNP sample, only (-)-epigallocatechin gallate (0.097% per sample amount), *trans*-cinnamic acid (0.025% per sample amount) and traces of caffeic acid were detected, whereas caftaric acid was not present in a measurable amount (Table 1; Figure 6A,C).

### 2.6. Relationship between the Investigated Parameters

Significant correlation, as estimated by Spearman’s correlation coefficient, has been recorded in all of the tested *P. oceanica’s* samples between (a) SOD and APX activity, and both enzyme activities and protein content (positive and negative correlation, respectively, *p* < 0.001); (b) CTCF value (i.e., H_2_O_2_ production) and both enzyme activities (negative correlation, *p* < 0.05) and CTCF value and protein content (positive correlation, *p* < 0.05); (c) values of bioactive assays: DPPH^•^-ABTS^•+^ (positive correlation, *p* < 0.05), DPPH^•^-OH^•^, DPPH^•^-O_2_^•−^, DPPH^•^-RP_0.5AU_, ABTS^•+^- O_2_^•−^, ABTS^•+^-RP_0.5AU_ and O_2_^•−^-RP_0.5AU_ (positive correlation, *p* < 0.01); and (d) phenolic content and values from all the bioactive assays (negative correlation, *p* < 0.05 or 0.01 or 0.001), with the exception of ABTS^•+^ (Table 2).

### 2.7. Antiproliferative Activity of Posidonia oceanica Extracts against Cancer Cells

**Figure 7:**
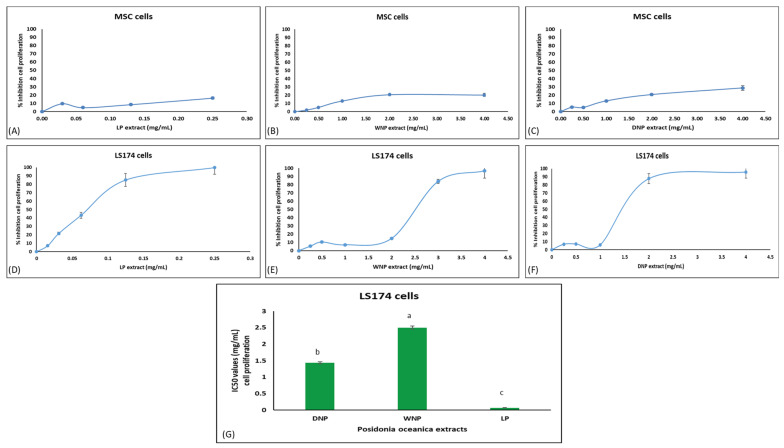
*Posidonia oceanica* extracts’ effects on cell proliferation, as estimated by XTT assay. Dose–response curves of MSC normal cells’ proliferation of (**A**) LP, (**B**) WNP and (**C**) DNP extract. Dose–response curves of LS174 cancer cells’ proliferation of (**D**) LP, (**E**) WNP and (**F**) DNP extract. (**G**) Extracts’ IC_50_ values against LS174 cell proliferation. The values are presented as the mean ± SD from at least three independent experiments. Different letters denote significantly different values between different extracts (*p* < 0.05).

*P. oceanica’s* extracts, apart from their antioxidant capacity, inhibited the proliferation of the LS174 colon cancer cells (Figure 7D–G). Specifically, the LP extract had IC_50_ value of 0.07 ± 0.007 mg/mL, while for WNP and DNP extracts, the values were 2.5 ± 0.048 and 1.4 ± 0.034 mg/mL, respectively (Figure 7G). LP extract had a significantly higher (*p* < 0.05) ability to inhibit the proliferation of the cancer cells as compared to both DNP and WNP extracts (Figure 7G).

Moreover, for examining if extracts’ inhibition of cancer cells’ proliferation was selective, their ability to inhibit proliferation of normal cells’, that is, human mesenchymal stem cells (MSC), was also examined (Figure 7A–C). The results showed that all the tested extracts exhibited lower inhibitory activity of cell proliferation in normal cells than in cancer cells (Figure 7A–E). Specifically, at the tested concentrations, the maximum inhibition of cell proliferation was 16.4% in MSC vs. 99.9% in LS174 for LP extract, 20.3% in MSC vs. 96.8% in LS174 for WNP extract, and 28.7% in MSC vs. 96.0% in LS174 for DNP extract (Figure 7A–E).

### 2.8. Quantification of the Phenolic Compounds Produced by Posidonia oceanica Dry ‘Necromass’ in the Wider Study Area—A Case Study

**Figure 8:**
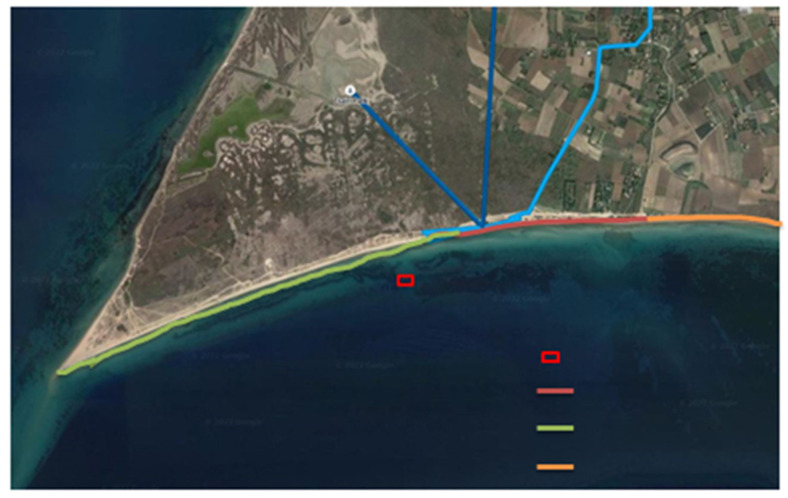
Map with the wider area of Potamos, Epanomi coast, Thermaikos Gulf (Aegean Sea). The map indicates the underwater collection station of *Posidonia oceanica* plants (rectangular frame) and the deposition areas of *P. oceanica’s* Dry ‘Necromass’ (DNP): the DNP deposition areas within the ‘Special Conservation Zone’ (SCZ) from where the DNP do not move away (green line); the deposition areas of DNP within the SCZ, from where the DNP is regularly removed offshore (red line) and the DNP deposition areas outside the SCZ zone (orange line).

In the broader area of Potamos, Emanomi Gulf, from the Cape Mytika up to the end of the Special Conservation Zone (SCZ) of ‘Natura 2000’ (demonstrated with green and red lines in Figure 8; see legend for details), the volume of *P. oceanica* DNP (without sand) deposited on the coast was calculated to be 1082.0 m^3^. The calculation of the DNP volume was based on the coastline length, where the DNP was deposited, as well as on the height (1–70 cm), width (5–40 cm) and depth (0 up to 20 cm) of DNP (Figure 1C). In addition, according to the Interreg Mediterranean, POSBEMED 2 program [8], the DNP volume outside the area of the SCZ (from where the dense *P. oceanica* meadow begins) (Figure 8, orange line) was estimated to be 1072.0 m^3^, and the DNP volume of the typical ‘*banquette*’ of the dense *P. oceanica* meadow (not shown in Figure 8) was estimated to be 5387.5 m^3^. Thus, the total volume of the DNP in the broader area of Potamos, Epanomi, the Thermaikos Gulf was calculated to be 7542 m^3^ (present study data and POSBEMED 2 data) [8], and the total dry mass was 1104.9 tons. Additionally, the volume of the WNP, which was transported by the wave action on the shores, ranged from 1600 to 11,200 cm^3^, and its mass from 119.9 to 216.9 g d.w.

Based on the volume and mass of *P. oceanica’s* DNP, in the broader area of Potamos at Epanomi coast, and on the yield (%) of the seagrass ‘Necromass’ extracts (11%), the total amount of the phenolic compounds that could be produced is 203.30 kg. Specifically, 119.11 kg of (-)-epigallocatechin gallate and 30.69 kg of *trans*-cinnamic acid could be produced. Furthermore, taking into account the management programs, according to which 20% of the produced seagrass dead mass was proposed to be utilized (see Section 1), 40.66 kg of the total phenolic compounds could be used for making high-added value products beneficial for human health, mainly consisting of 23.82 kg of (-)-epigallocatechin gallate and 6.14 kg of *trans*-cinnamic acid.

## 3. Discussion

Seagrasses are an important source of bioactive metabolites, with antioxidant, anticancer, antibacterial, antifungal and antihypertensive activities [10,50,51,52]. Although these activities have not been systematically investigated, due to the great seagrass biodiversity [13], bioactive compounds have been isolated from several seagrass species (see review articles written by Zamani et al. [10] and Kim et al. [40]), including *Posidonia oceanica* [31,32,33,34,35,39,40] (Table S2). Some of seagrasses’ compounds exhibit antioxidant properties. For example, fibers, fatty acids (e.g., uronic acid and oleic acid), proteins, carbohydrates (e.g., galactose, ramose, arabinose, fucose, mannose, lactose, xylose, maltose), minerals, phenolic compounds, flavonoids, tannins and vitamins C and E have been shown to possess antioxidant properties [16,33,40,50,52,53,54].

Biotic and abiotic environmental factors [2,3,20,21,22,23,55,56,57,58,59] can cause oxidative stress to seagrasses [24,25,26,27]. Oxidative biomarkers have been proposed as suitable for assessing the physiological state of these organisms [24,60]. However, as *P. oceanica* plants grow at great depths (10–50 m), they are not expected to be exposed to a wide range of environmental changes (e.g., temperature, drought, UV radiation, toxic pollutants) compared to shallow water seagrass species such as *Cymodocea nodosa* [2,57,58]. Although *P. oceanica,* in the study area, is protected by the ‘Natura 2000’ convention, its shoot density (211–409 shoots m^−2^), according to ‘POSBEMED 2’ [8], has been ranged from irregular (<237) to sub-normal (<349) [61], which may indicate possible environmental stress, contributing, among other effects, to increased ROS production [24,25,26,27,60]. ROS are derivatives of anaerobic plant metabolism, which, at low concentrations, participate in physiological cell procedures, while, in excess, they can cause damage to the macromolecules (e.g., lipids, proteins, DNA) [20]. Seagrass cells may activate antioxidant mechanisms, which may (a) neutralize free radicals, acting as H^+^ or e^-^ donors or acting directly on them; (b) form ion chelates with transition-metallic elements; (c) prevent the production of free radicals by increasing antioxidant enzymes’ activity (e.g., SOD, CAT, GPX); and (d) repair or remove the affected molecules [3,25,26,27,29,35,60,62]. In our study, the antioxidant efficacy of *P. oceanica* leaves to counteract oxidative stress, under field conditions, seems to be relatively low. Specifically, in LP and WNP samples, antioxidant enzymes (i.e., SOD and APX) were activated, but not to a great degree that could counteract oxidative stress (i.e., H_2_O_2_ production), since CTCF values were maintained at high levels. *P. oceanica* is a stenohaline and stenothermal species that grows in high depths and has lower leaf growth rates, shoot turnover, plasticity and response to stress, compared to fast-growing seagrass species, which are found to shallow water and have adapted to environmental changes (e.g., *Cymodocea nodosa*) [57,63,64]. In a previous study, *P. oceanica* has been demonstrated to have lower antioxidant response compared to the shallow water seagrass species [57]. In particular, *P. oceanica* had lower expression of genes encoding antioxidant enzymes catalyzing the conversion of the superoxide radical (O_2_^•−^) to hydrogen peroxide (H_2_O_2_), recovery genes involved in the reduction of H_2_O_2_ to hydroxyperoxides (e.g., genes encoding CAT, APX and GPX synthesis) and dsp5 gene leading to cell apoptosis [57]. Genes related to SOD production in chloroplasts (FSD genes) and mitochondria (MSD genes) play an active role in protecting cells from oxidative stress [65]. Moreover, in a previous study, *P. oceanica* plants of the intertidal zone demonstrated higher CAT, SOD and GPX activity and greater lipid peroxidation, due to oxidative stress, compared to its plants growing in greater depths, as in our research (11 m depth) [60].

The methanol extraction’s yield (24%) of LP leaves was similar to those in other marine macrophytes, whether they were treated with the same or different extraction method [66,67,68,69]. However, the yield of ‘Necromass’ samples (9% in WNP and 11% in DNP) was lower than that in LP samples, which may be due to cellular components loss because of the deposition of plant material on the shores.

The high H_2_O_2_ levels in LP leaves justify the high phenolic content in LP leaf extracts. Namely, as a response to oxidative stress induced by the high H_2_O_2_ level, the *P. oceanica* leaves produce phenols which, in turn, contributed to the LP extracts’ high free radical scavenging (i.e., low IC_50_ values) against DPPH^•^, ABTS^•+^, O_2_^•−^ and OH^•^ radicals, as well as to their high reducing power. LP extracts exhibited high DPPH^•^ scavenging activity (IC_50_ value: 8.2 μg/mL) similar to other studies [11], although higher IC_50_ values of *P. oceanica* leaf extracts have also been reported (e.g., IC_50_: 32.00 μg/mL) [46]. These differences may be attributed to different extraction methods and solvents [46]. Moreover, the *P. oceanica* extracts’ scavenging capacity against DPPH^•^ was considerably higher than those in other seagrass species, processed by various analytical methods, such as *Cymodocea serrulata* (IC_50_: 44.47 μg/mL) [70], *C. rotundata* (IC_50_: 123.72 μg/mL; 125.8 μg/mL [71,72] and *C. nodosa* (IC_50_: 1220 μg/mL) [51]. Finally, *P. oceanica’s* capacity to scavenge DPPH^•^ radical was much higher than those (IC_50_: 1400–79,000 μg/mL) reported in one of our previous studies for thirteen seaweed species collected again from the Thermaikos Gulf [69], and for seaweed species from other areas (IC_50_: 204.08–500.00 μg/mL [73]; 812.22 μg/mL [74]; 5000–15,940 μg/mL [75]; 142.5 μg/mL [76]; 97–410 μg/mL [77]). Some of the above seaweeds are exploited for their antioxidant properties, due to their content in bioactive components, for instance polyphenols and bromophenols [78,79]. The ABTS^•+^ free radical scavenging of the LP extracts (IC_50_: 1.6 μg/mL) was much higher than that recorded for the marine angiosperms *Cymodocea* spp. and *R. maritima* [51,52,70,72], as well as higher than fourteen seaweed species treated with the same procedure as in this study (IC_50_ from 20 to 15,000 μg/mL [69]) or with other methods [68,69,77]. The OH^•^ radicals are the most harmful of the ROS species, since they oxidize too fast all biological macromolecules [80]. The OH^•^ assay’s IC_50_ values in the LP and WNP extracts (IC_50_: 267 and 416 μg/mL, respectively) were similar to those recorded in the same seagrass species, after 80% *v*/*v* methanol extraction for free (IC_50_: μg/mL) and bound (IC_50_: 276 μg/mL) phenolic compounds [35]. The scavenging capacity against O_2_^•−^ of *P. oceanica* extracts (IC_50_: 71.0 μg/mL) was similar to that found in the same species previously (IC_50_: 66.80 μg mL^−1^ for free phenolic compounds) [35], but also to those in the rhodophyte *Gigartina* sp. (IC_50_: 50–70 μg/mL). However, *P. oceanica* extracts’ scavenging activity against O_2_^•−^ was much higher than those in other seaweed species (ΙC_50_: 140–6400 μg/mL) [69]. The reducing power of LP extracts (RP_0.5AU_: 19.5 μg/mL) was about half of that observed in other studies in 80% *v*/*v* methanol extracts (RP_0.5AU_: 8.73 μg/mL for free phenolic compounds and 9.40 μg/mL for bound phenolic compounds [35]). However, LP extracts’ reducing power was much higher than that of fourteen seaweed species (RP_0.5AU_: 240–15,000 μg/mL) [69].

Non-enzymatic antioxidants (e.g., lignin, tannins, phlorotannins, steroids, vitamin C and E, phenols and flavonoids) can be produced in all plant organs, as products of secondary metabolism, contributing to their antioxidant and/or anticancer activities [51,79,81]. In fact, in marine and freshwater macrophytes, a significant relationship has been recorded between their antioxidant activity and phenolic content [32,69,82]. The high TPC in the LP leaves (222.8 mg g^−1^ d.w.) supported their extracts’ ability to scavenge free radicals, as shown by the negative correlation between antioxidant assays’ values of extracts and TPC. It should be noted that TPC in LP extracts was evaluated in a relatively limited number of studies [43,55,83,84,85,86]. Relatively lower TPC in the adult leaves of *P. oceanica* (55.5–94.9 mg g^−1^ d.w.) [83] and their rhizomes (7.1–35.2 mg g^−1^ d.w. [55]; 26.2–28.9 and 22.31–27.62 mg g^−1^ d.w. [84,85]) were recorded, compared to other LP leaves (second adult and intermediate leaves) in our research. Similarly, lower TPC was recorded in the *P. oceanica’s* intermediate leaves (45.4 mg g^−1^ d.w.), adult leaves (32.0 mg g^−1^ d.w.) and sheaths (16.8 mg g^−1^ d.w.) [43], compared to our LP values. Moreover, in eight other seagrass species, a comparatively lower TPC (0.334–1.399 mg g^−1^ d.w) was recorded [32]. The TPC may differ across organisms, populations, organs and tissues [40,45] and is, in fact, influenced by the plant growth conditions and physiology [87]. For example, higher TPC was recorded in the distal section of *P. oceanica* rhizomes than in its leaves, which was attributed to the longer lifespan of the rhizomes and, consequently, to their exposure to various types of environmental stresses, as well as to the faster rate of leaf turnover [55]. Moreover, TPC can vary depending on the collection period. For example, TPC is elevated during summer, due to the creation of new plant cells [88]. Thus, since in the present study, *P. oceanica* was collected in April, a relatively higher TPC content is expected during the summer months. However, in another research study, *P. oceanica’s* phenolic content was higher during winter months [83,86]. Moreover, TPC of *P. oceanica’s* extracts was much higher than that found in seaweed’s extracts in different studies (0.55–65.00 mg GAE/g d.w.) [67,68,75,89,90,91,92]). Finally, the phenolic compounds in marine angiosperms have been proposed as early warning biomarkers for the biomonitoring of coastal systems [27,30,84,85,93].

Thirty seven phenolic compounds have been determined, so far, in LP’s extracts (see Table S2). In the present study, 10 out of the 17 examined phenolic compounds were quantified, while 2 of them, (-)-epigallocatechin gallate and rutin hydrate, were determined for the first time in *P. oceanica* extracts. The analysis of the individual phenolic composition of the LP leaf extracts showed that they contained high concentrations of chicoric acid (70,590 μg g^−1^ d.w.), and at lower levels epigallocatechin gallate (39,330 μg g^−1^ d.w.), *p*-coumaric acid (19.160 μg g^−1^ d.w.), rutin hydrate (3910 μg g^−1^ d.w.), sinapinic acid (1890 μg g^−1^ d.w.) and caftaric acid (1620 μg g^−1^ d.w.), ferulic acid (92 μg g^−1^ d.w.) and *trans*-cinnamic acid (38 μg g^−1^ d.w.). Chicoric acid having immunostimulant activity promotes phagocyte activation, in vitro and in vivo, and inhibits hyaluronidase function [94]. Additionally, chicoric acid exhibits antiviral activity by inhibiting HIV integrase [95] and herpes virus levels [96], and has antioxidant activity [97]. Similarly, Grignon-Dubois & Rezzonico [98] found in *P. oceanica* plants that chicoric acid was the most abundant phenolic compound (2490–12,783 μg g^−1^ d.w.), reaching 87–96% of total polyphenols and followed by caftaric acid (530–13,807μg g^−1^ d.w.). Moreover, other studies have reported *P. oceanica’s* methanolic or ethanolic extracts to contain some of the compounds found in the present research, but at much lower concentrations (e.g., chicoric acid: 4.39–4991.81 μg g^−1^ d.w.; *p*-coumaric acid: 6.75–150.99 μg g^−1^ d.w.) [17]. Furthermore, Haznedaroglu & Zeybek [44] found chicoric acid (138.6 and 38.4 μg g^−1^ d.w.), coumaric acid (4.2 and 2.4 μg g^−1^ d.w.) and *trans-*ferulic acid (2.2 and 1.5 μg g^−1^ d.w.) in the young and adult leaves of *P. oceanica*, respectively, and vanillin and catechin at relatively lower amounts. Additionally, Agostini et al. [43] reported 3,5-dimethyl-4-hydroxycinnamic acid at concentrations of 300 to 800 μg g^−1^ d.w. in samples from different areas. In *P. oceanica* plants, under biotic stress, *trans*-ferulic acid, phloridzin, phloroglucinol, *p*-anisic acid, acetosyringone, sinapic acid, phenol, *p*-hydroxybenzoic acid, *p*-coumaric acid and cinnamic acid were produced, which represent the 95% of the total phenolic content [49]. Synopsis of phenolic compounds detected in the seagrass *Posidonia oceanica* is given in Table S2.

The overproduction of the reactive radicals OH^•^ and O_2_^•^ is associated with carcinogenesis [99,100]. Thus, apart from antioxidant activity, LP extracts inhibited LS174 colon cancer cell growth (IC_50_: 70.0 μg/mL). Importantly, all the three tested extracts exhibited selective activity in LS174 cancer cells, since their antiproliferative activity was higher in cancer cells than in normal cells. Our results were similar to that demonstrated in the same cancer cells by another study for a *P. oceanica’s* extract prepared by supercritical extraction (IC_50_: 60 μg/mL) [49]. However, an ethanolic extract of *P. oceanica* leaves exhibited higher IC_50_ value (IC_50_: 101 μg/mL) than our value, but in the HT-29 colon cancer cell line [49]. Furthermore, the same study [49] showed that both extracts could inhibit breast (MCF-7, MDA-MB-231, SK-BR-3), cervical (HeLa) and prostate (PC-3) cancer cells with IC_50_ values ranging from 53 to 464 μg/mL. According to Selvimli-Gur et al. [49], the antiproliferative activity of the extracts was partly due to their phenolic compounds, such as *p*-coumaric, rosmarinic, chicoric acid, benzoic acid, caffeic acid and *trans*-ferulic acid. Other studies have shown that ethanolic extract of this species inhibited through autophagy the migration of HT-1080 epithelial cells, which indicated antimetastatic activity [101]. Moreover, the hydroalcoholic extract of *P. oceanica* leaves, which are rich in polyphenols and carbohydrates, inhibited SH-SY5Y neuroblastoma cell migration [48]. Previous studies have also reported the ability of various seagrasses (e.g., *Thalassodendron ciliatum*, *Thalassia testudinum* and *Cymodocea nodosa*) to inhibit the growth of a variety of cancer cells being different than our cells, such as lung NSCL-N6 and A549, cervical HeLa, myeloid leukaemia K562, breast MCF-7 and liver HepG2 cancer cells, with IC_50_ ranging between 13.28 and 500 μg/mL [51,52,54,74,102,103,104,105]. It should be noted that apart from LP extracts, WNP and DNP extracts also exhibited inhibition against LS174 colon cancer cells, suggesting that *P. oceanica’s* ‘Necromass’ could be exploited for the development of products with antiproliferative properties against cancer cells.

Given the ecological role of *P. oceanica* in the Mediterranean Sea [1,2,3,4,21,22,56]) and the rapid decline of its meadows [6,106]), the latter are considered to be priority habitats (1120*, Directive 92/43/EEC, NATURA 2000 Network) and are protected by international conventions. Provided that there is an acceptable management plan for *P. oceanica* meadows, which includes either the collection of a sufficient amount of living leaves, without adverse effects on them, and/or the cultivation of *P. oceanica* plants in the field, our research demonstrated that the LP leaves could be exploited for their antioxidant activity and antiproliferative properties against cancer cells, and the development of high-added value products containing phenolic compounds and promoting human health. It is remarkable that the research interest has turned recently to the utilization of seagrass dead mass on the shores for the production of useful products for human health, in the case that they retain the properties of LP leaves after desiccation [16,17,98,106].

The reduction of protein content of the DΝP, compared to the WNP material and LP leaves, may indicate an inhibition of protein biosynthesis and/or acceleration of protein decomposition [25,26,29], possibly due to leaf desiccation on the shores [62]. In the study from Benito-González et al. [16], the protein content of *P. oceanica* ‘Necromass’ ranged from 58.4–363.0 mg g^−1^, depending on the extraction method. At the same time, the higher SOD and APX activities (U mg^−1^ protein) in the DNP, compared to the WNP sample and the LP leaves, seem to neutralize (i.e., low CTCF values in DNP) a significant part of the H_2_O_2_ produced by the LP leaves, during plant desiccation in the shores. Subsequently, the low H_2_O_2_ production may account for the lower DNP extracts’ ability to scavenge free radicals, compared to LP leaves.

Regarding the determination of antioxidant activity in *P. oceanica* dead leaves, there have been two previous studies [16,17]. However, these studies concerned dead leaves with different stages of desiccation than that of our research. Specifically, these studies were on either dead leaves from shores, without determining the stage of desiccation [16], or on naturally detached foliar bundles, which have not yet been deposited on the shores [17]. However, like our study, Messina et al. [17] recorded that ‘brown’ leaf (i.e., photosynthetically inactive) extracts prepared by 70% *v*/*v* ethanol of *P. oceanica’s* detached leaves showed lower scavenging activity against DPPH^•^ radical, compared to the dead mass consisting of ‘green’ leaves being cut from the plant due to storms, but not deposited on the shores. Even the extracts from detached leaves, consisting of ‘half green’ leaves exhibited higher antioxidant activity than the ‘brown’ leaf extracts [17]. Interestingly, all detached leaf extracts examined by Messina et al. [17] in DPPH assay had lower activity (IC_50_ values: 8000–14,530 μg/mL) than our dead mass extracts (IC_50_ 2850 μg/mL in WNP and 1600 μg/mL in DNP). Despite the lower DNP’s antioxidant activity, compared to that of LP leaves, DNP antioxidant capacity remained higher than most of the seaweed species’ antioxidant activity, collected from Thermaikos in one of our previous studies [69].

The evaluation of the TPC in the LP extracts helped to determine the loss of TPC according to leaves’ desiccation stage (i.e., WNP or DNP) after their detachment from the plant. Although seagrasses are considered highly resistant to decay [107] and their TPC persist during their deposition on shores [108], the present study showed a significant loss (from 100 to 120-fold) of the TPC, during the seagrass deposition on the coasts, in both DNP and WNP materials. The decrease in the TPC in the DNP material may be due to the reduction of the number of individual phenolic compounds (8 compounds in LP vs. 3 compounds in DNP) and/or in their amount, during their desiccation in the shores. Specifically, after the deposition of *P. oceanica* shoots on the shores (i.e., DNP sample), only small amounts of (-)-epigallocatechin gallate (970 μg g^−1^ d.w.) and *trans*-cinnamic acid (250 μg g^−1^ d.w.), and traces of caffeic acid remained. According to Dumay et al. [83], caffeic acid and cinnamic acid were detected in *P. oceanica* ‘Necromass’. In addition, previous studies on *P. oceanica* ‘Necromass’ have recorded chicoric acid (2490–12,110 μg g^−1^ d.w.), *trans*-ferulic acid (300–800 μg g^−1^ d.w.) and caffeic acid (530–1380 μg g^−1^ d.w.) [42,98,109]. Moreover, nine natural phenolic compounds have been shown to remain in *P. oceanica* extracts from detached leaves [17]. Messina et al. [17] found that *P. oceanica* detached leaf extracts, consisting of ‘green’ leaves being still photosynthetically active, showed higher TPC (0.019 mg GAE g^−1^) than the extracts from detached leaves, consisting of ‘half green’ or ‘brown’ (i.e., photosynthetically inactive) leaves. Specifically, in detached leaves consisting of ‘green leaves’, high concentrations of *p*-hydroxybenzoic acid (208.7 μg g^−1^ d.w.), gallic acid (103.2 μg g^−1^ d.w.), chicoric acid (48.7 μg g^−1^ d.w.) and at smaller concentrations of caffeic, *trans*-ferulic, vanillic, *p*-coumaric acid and quercetin were measured [17]. In fact, a reduction of the above compounds by 8.5-fold in detached leaves consisting of ‘half green’ leaves and by 4-fold, in detached leaves consisting of ‘brown’ leaves were recorded [17]. In the present study, it was found that during the seagrass deposition on the coasts, there was higher loss (i.e., from 100 to 120 fold) of the TPC and fewer individual phenolic compounds (i.e., 3 compounds identified), compared to the study of Messina et al. [17] (i.e., from 4 to 8.5-fold loss of the TPC; 9 compounds identified). These differences may be due to the different stage of leaf desiccation among the studies. Namely, Wet ‘Necromass’ deposited on the shores at the sea level (WNP) and beach casts and ‘*banquettes*’ (DNP) were collected in our study, while detached leaves not yet deposited along the shores were collected from Messina et al. [17].

Given the ecological value of *P. oceanica* meadows in the Mediterranean Sea [4,5], their ‘*banquettes*’ are also considered as priority habitats. However, there is still no legislative framework in Mediterranean countries for the removal and management of its beach casts and ‘*banquettes*’. Thus, they are treated either as important resources or as waste. At the initiative of the municipal authorities, they are systematically removed from the coasts and disposed untreated in the landfills at a high cost, although there is a directive of the European Union, stating that the sanitary landfill of slurries without treatment and recycling is not allowed [9,41]. Ιn these ecosystems, management plans with low ecological impact, such as partial removal, that is, the mass located in the first 10 cm of the beach casts’ area or removal of only 20% of their beach casts [8,9] have been proposed. As previously mentioned, despite the decrease in the TPC content in the DNP sample, it is retained the antioxidant and antiproliferative properties of LP sample. Moreover, our study suggested that the wider area of Potamos, the Epanomi coast (the Thermaikos Gulf, the Aegean Sea) can produce 203.30 kg of phenolic compounds, including 119.11 kg of (-)-epigallocatechin gallate and 30.69 kg of *trans*-cinnamic acid, if the total mass of DNP material (i.e., 1104.9 tons) is used. Alternatively, if the management plan is taken into account, suggesting removal of 20% of the produced dead mass, 40.66 kg of polyphenolic compounds will be produced, including 23.82 kg of (-)-epigallocatechin gallate and 6.14 kg of *trans*-cinnamic acid.

## 4. Materials and Methods

### 4.1. Collection of Posidonia oceanica Samples

For the selection of the study area (Potamos, Epanomi, the Thermaikos Gulf, Northern Aegean Sea) (40°37.81′11″ 38°43.64′96″ N, 22°91.25′44″ 32°29.44′77″ E) (Figure 8), previous surveys were taken into account: (a) the current state of marine habitat types 1120* of Directive 92/43/EEC (Ministry of Health and Welfare 2001); (b) the mapping of *P. oceanica* meadows during 2012, in the framework of “Actions for the conservation of coastal habitats of the NATURA 2000 network, in the Epanomi and Angelochori’ lagoons–LIFE09 NAT/GR/000343, ACCOLAGOONS, 2012, Action A2” [110]; and (c) the framework of the project “Governance and management of *Posidonia* and dune systems in the Mediterranean Sea” (Interreg Mediterranean-POSBEMED 2, Action 3.3 2021) [8].

For the underwater research, during the beginning of April 2022, 84 orthotropic *P. oceanica* rhizomes with their leaf bundles were selected, by scuba-diving, at about 11 m depth, from the coastal area of Potamos, Epanomi, within the limits of the Special Conservation Zone (SCZ zone 1220012) of the ‘Natura 2000’ area (Figure 8). The samples were transported to the laboratory in vessels containing seawater. In 15 leaf shoots, the following phenological parameters were measured: number of leaves per bundle in each of the leaf-age classes (adult, intermediate and young leaves, Figure 1A) [2] and the leaf length and width, and their leaf areas. From 140 leaf shoots, in each leaf class, the wet and dry leaf biomass (at 50 °C up to constant weight) and their mass loss (%) were calculated.

After completing their life cycle, the leaves of the seagrass (life span 4–13 months), fall and deposit in the sediment and along with rhizome remains form the beach casts/’banquette’, which are termed ‘Necromass’ [111]. Part of the leaf dead mass is transported by the waves and is initially deposited on the coast at the sea level where the waves break onto the beach (Figure 1B); in the present study, this plant material is termed as Wet ‘Necromass’ (WNP). An estimation of the WNP’s volume was performed by recording their height (i.e., the distance from the coast to their highest point) in ten areas of 40 × 40 cm each (Figure 1B). Moreover, the Dry ‘Necromass’ (DNP) was collected, that is, the brown-coloured ‘Necromass’ that is deposited at higher points in the coastal area and formed piles mixed with sand (i.e., *’banquettes’*) (Figure 1C). The WNP and DNP materials were collected from the area within the SCZ zone, where no mass removal practices by humans are applied (demonstrated by the green line in Figure 8).

Subsequently, for the calculation of the total volume and mass of the DNP in the broader collection area of Potamos, the Epanomi coast (case study), i.e., within the SCZ zone of ‘Natura 2000’, where the DNP is not removed (Figure 8, green line, data of the present study), and within the SCZ zone, where the DNP is regularly removed (Figure 8, red line, data of the present study), the following was measured: (a) the length of the coastline, where DNP is deposited; (b) the width of DNP, i.e., the distance between the lowest and highest point of DNP deposition on the shore; (c) the depth below the sand; and (d) the height they occupy from the coast level (Figure 1C). Additionally, the DNP’s volume outside the SCZ zone (Figure 8, orange line) and mass in the area of the typical *P. oceanica* meadows, creating the well-known ‘*banquettes*’ (not shown in Figure 8) (data of POSBEMED 2 [8]), were taken into account for the total measurements of DNP that can be produced in the ‘case study’. The wet and dry mass of all *P. oceanica* samples (after drying at 50 °C), as well as their weight loss (%), due to the removal of water, was also calculated.

### 4.2. Imaging of Hydrogen Peroxide Production in Living Leaves (LP) of Posidonia oceanica

After the collection, the LP leaves without epiphytes (i.e., 2nd adult leaf and intermediate leaf from n = 9 shoots) and the DNP and WNP necromass’ materials (n = 9 pieces each) were incubated with 2′,7′-dichlorofluorescein diacetate (DCF-DA) in dimethyl sulfoxide (DMSO) after 4–48 h and observed under a Zeis AxioImager Z.2 fluorescence microscope equipped with an MRc5 Axiocam [27,29,111]. The fluorescence intensity of the cells was measured using ImageJ version 1.54 software (U. S. National Institutes of Health, Bethesda, MD, USA). The corrected total cell fluorescence (CTCF) was calculated as the following:

CTCF = integrated density − (area of selected cell × mean fluorescence of background readings)(1)

The mean CTCF values were derived from approximately 162 measurements for living leaves (three regions per three segments (i.e., apex, middle, base) of each leaf; n = 18 leaves), and from 27 measurements for ‘Necromass’ samples (three regions per leaf piece; n = 9 leaf pieces) [27,29,112].

### 4.3. Assessment of Antioxidant Enzymes in LP, WNP and DNP Samples and Protein Content

Oxidative stress responses were assessed in LP leaves (second adult and intermediate leaf in a ratio: 1/2.7), and in WNP and DNP samples, by measuring the activity of the antioxidant enzymes superoxide dismutase (SOD) and ascorbate peroxidise (APX) and the total protein contents. Three samples (100 mg wet weight) of each plant category were grounded in liquid nitrogen, followed by deep-freezing storage (−80 °C). In each sample, three subsamples were taken and homogenized in 3 mL of 50 mM of sodium phosphate buffer (pH 7.8), containing 0.1 mM of ethylenediaminetetraacetic acid (EDTA) and 2% *w*/*w* of polyvinylpolypyrrolidone (PVPP), and then, the subsamples were centrifuged at 16,500× *g* for 30 min at 4 °C. The samples’ protein content was used to normalize the values. The protein content was determined as previously described [113]. Specifically, 50 µL of each sample was added to 5 mL of a solution containing 100 mg of Coomassie Brilliant Blue, and 50 mL of 95% *v*/*v* ethanol (Sigma), 100 mL of 85% *w*/*v* ortho-phosphoric acid and distilled water were added (protocol of Bradford [113]). The optical absorbance was measured at 595 nm with a UV-1700, Shimadzu Spectrophotometer (Tokyo, Japan) [25,26,27,28,29]. The protein content (mg g^−1^ww) was calculated based on a standard curve of bovine serum albumin.

SOD activity (U mg^−1^ protein) was measured according to Beyer and Fridovich method [114]. Specifically, 50 µL of each subsample (3 subsamples/sample) was added to 5 mL of a solution containing 50 mM potassium phosphate buffer (pH 7.8), 0.1 mM ethylenediaminetetraacetic acid (EDTA, Applichem), 0.025% (*v*/*v*) (2-[4-(2,4,4-trimethylpentan-2-yl)phenoxy] ethanol, TritonX-100), 13 mM methionine, 0.075 mM nitro blue tetrazolium chloride (NBT) and 0.002 mM riboflavin (riboflavin, 98%). Then, SOD activity was detected at 560 nm using a Spectrophotometer (PharmaSpec UV-1700, Shimadzu, Tokyo, Japan), according to the inhibition rate of nitro blue tetrazolium (NBT) to photochemical decline, as previously described [25,26,27,29].

Ascorbate peroxidase (APX) activity (U mg^−1^ protein) was determined according to Nakano and Asada [115]. Specifically, 50 µL of each subsample (3 subsamples/sample) were mixed with 5 mL of a reaction solution containing 50 mM potassium phosphate buffer, 0.1 mM ethylenediaminetetraacetic acid (EDTA) (pH 7.8), 0.5 mM ascorbic acid and 0.1 mM H_2_O_2_. APX activity was determined, as previously described, by the alteration in the optical absorbance due to oxidation, at 290 nm for 1 min, using a Spectrophotometer (PharmaSpec UV-1700, Shimadzu, Tokyo, Japan) [25,26,27,29]). All the measurements were performed in triplicate.

### 4.4. Preparation of Posidonia oceanica Extracts

The extracts from LP, WNP and DNP samples were prepared as previously described [69]. In brief, to prepare the extract, the P. oceanica samples were ground and soaked in a solution of 80% *v*/*v* methanol (1:30 dry weight sample to solvent volume). The solution was then subjected to sonication using an UP400S Hielscher sonicator (Teltow, Germany) for 20 min at 20 cycles and 70% amplitude. Afterwards, the solution was placed in a shaker incubator (Innova^®^ 40, New Brunswick Scientific; St Albans, UK) at 25 °C and 150 rpm for 48 h. Subsequently, the extract solution was filtered using a 0.45 μm Whatman filter paper. The solvent was removed through rotary evaporation (IKA, Werke RV-06-ML; Staufen, Germany) at 30 °C and 150 rpm under reduced pressure, followed by freeze drying (CoolsafeTM, Scanvac; Allerod, Denmark) for 24 h, resulting in the extracts in powder form.

The weight of the dried powder was measured to determine the percentage yield of the extraction process, using the following equation:

Extraction yield (%) = [dry extract (g)/dry seaweed (g)] × 100(2)

The extracts were kept at −20 °C until further use.

### 4.5. Phenolic Content

In extracts from all *P. oceanica* samples (i.e., LP, WNP and DNP), the total phenolic content (g d.w.) (TPC) was evaluated spectrophotometrically at 765 nm, by using the Folin–Ciocalteu reagent as described previously [116]. The TPC was determined by constructing a standard curve that correlated absorbance values with known concentrations (ranging from 50 to 1500 μg/mL) of gallic acid. The TPC was expressed as mg of gallic acid equivalents (GAE) per g of dry weight (d.w.) of extract. At least three independent measurements were performed for each sample.

The individual phenolic compounds in LP and DNP samples were determined by UHPLC-DAD analysis as described previously [69]. Specifically, ECS05 UHPLC-DAD equipment (Prague, Czech Republic) consisting of a quaternary gradient pump (ECP2010H) and a gradient box with degasser (ECB2004) was employed coupled with a diode array detector (ECDA2800 UV-Vis PDA Detector). Chromatographic separation of the constituents was implemented on a Fortis Speed Core column (C18, 2.6 μm, 100 × 4.6 mm) (Cheshire, UK) at 25 °C. An aqueous mobile phase acidified with 0.1% formic acid (A) and methanolic one (B) was utilized at a total flow rate of 1 mL/min. The elution gradient at t = 0 min was 90% A, remained constant for 5 min, while at 8.5 min it was set to 72% A, at 30 min to 40% A and then remained constant for 3 min. After each run (10 μL injection volume), equilibration was performed for 3 min at the initial conditions. Detection of the constituents was recorded at 280, 270, 328 and 318 nm. Data were processed by using Clarity Chromatography Software v8.2 (DataApex Ltd., Prague, Czech Republic). Identification and quantification of the individual phenolic compounds in all samples was based on the following mixture of standards: gallic acid, p-hydroxy-benzoic acid, vanillic acid, caffeic acid, *p*-coumaric acid, *trans*-ferulic acid, rutin hydrate, *trans*-cinnamic acid, chicoric acid, caftaric acid, (-)-epigallocatechin gallate, chlorogenic acid, myricetin, sinapinic acid, quercetin, 4′,5,7-trihydroxyflavone and hesperidin. Subsequently, the standards were diluted in methanol, at a range of 0.78–200 mg L^−1^ to construct each calibration curve and analyzed as mentioned above, at 280, 270, 328 and 318 nm. Analyses of the phenolic content were carried out in all extracts at 7000 ppm methanol.

### 4.6. 2.2-Diphenyl-Picrylhydrazyl (DPPH^•^) Radical Scavenging Assay

DPPH^•^ assay was performed as described previously [69]. In brief, 1.0 mL of freshly prepared methanolic solution of the DPPH^•^ radical (at 100 μM) was mixed with each tested extract dissolved in distilled H_2_O at different concentrations. These concentrations were from 0.002 to 0.068 mg/mL for LP extract, from 0.25 to 4 mg/mL for WNP extract and from 0.125 to 2 mg/mL for DNP extract. The contents were vigorously mixed, incubated at room temperature under dark for 20 min and its absorbance values were measured at 517 nm. The measurement was conducted on a Perkin Elmer Lambda 25 UV/VIS spectrophotometer (Waltham, MA, USA). In each experiment, a negative control was included, which consisted of the tested sample alone in methanol. Ascorbic acid was used as positive control. The percentage of radical scavenging capacity (RSC) of each tested extract was calculated according to the following equation:

Radical scavenging capacity (%) = [(A_control_ − A_sample_)/A_control_] × 100 (3)

At least three independent experiments were performed for each sample.

### 4.7. 2.2-Azino-bis(3-ethylbenzthiazoline-6-sulfonic acid) (ABTS^•+^) Radical Scavenging Assay

Each extract was examined in ABTS^•+^ radical scavenging assay as previously reported [116]. In brief, ABTS^•+^ radical was produced by mixing 2 mM ABTS with 30 μM H_2_O_2_ and 6 μM horseradish peroxidase (HRP) enzyme in distilled water (1 mL). The solution was vigorously mixed, and incubated at room temperature under dark for 45 min until ABTS^•+^ radical was formed. Then, 10 μL of each extract concentration in aqueous solution were added in the reaction mixture and the absorbance at 730 nm was measured. The extract concentrations were from 0.3 to 8.5 μg/mL for LP extract, from 0.125 to 2 mg/mL for WNP extract and from 0.063 to 2 mg/mL for DNP extract. In each experiment, an aqueous solution of the tested sample containing ABTS^•+^ and H_2_O_2_ was used as blank, while the ABTS^•+^ radical solution with 10 μL H_2_O was used as control. The percentage of radical scavenging capacity (RSC) of the tested extracts was calculated as mentioned above for the DPPH assay. Ascorbic acid was used as positive control. At least three independent experiments were performed for each tested compound.

### 4.8. Hydroxyl Radical (^•^OH) Scavenging Assay

The hydroxyl radical (OH^•^) scavenging activity was carried out according to the method of Kerasioti et al. [117]. In brief, 75 μL of aqueous solution of extracts at different concentrations were added to a mixture consisting of 450 μL sodium phosphate buffer (0.2 M, pH 7.4), 150 μL 2-deoxyribose (10 mM), 150 μL FeSO_4_-EDTA (10 mM), 525 μL H_2_O and 150 μL H_2_O_2_ (10 mM), followed by incubation at 37 °C for 4 h. Then, 750 μL TCA (2.8%) and 750 μL 2-thiobarbituric acid (1%) were then added and the samples were further incubated at 95 °C for 10 min. The extract concentrations in this assay were from 0.004 to 1.0 mg/mL for LP extract, from 0.25 to 8 mg/mL for WNP extract and from 0.125 to 4 mg/mL for DNP extract. The samples were cooled on ice for 5 min and centrifuged at 3000 rpm for 10 min at 25 °C. The absorbance was measured at 520 nm. Negative controls were included in each experiment, where samples without H_2_O_2_ were used. The samples without extract were used as negative controls. Ascorbic acid was used as positive control. The OH^•^ radical scavenging activity was calculated according to the equation:

% OH^•^ radical scavenging activity = [(Abs_control_ − Abs_sample_)/Abs_control_] × 100 (4)

where *Abs*_control_ and *Abs*_sample_ are the absorbance values of the control and the tested sample, respectively. At least three independent experiments were performed for each tested compound.

### 4.9. Superoxide Anion Radical (O2^•−^) Scavenging Assay

The superoxide anion radical (O_2_^•−^) scavenging activity of the extracts was evaluated as described previously [69]. In brief, O_2_^•^ are produced by the PMS-NADH system through oxidation of NADH following the reduction of nitroblue tetrazolium (NBT) and are measured spectrophotometrically at 560 nm. Antioxidants may scavenge O_2_^•−^, and consequently reduce absorbance. The tested concentrations in this assay were from 0.034 to 1.0 mg/mL for LP extract, from 0.125 to 4 mg/mL for WNP and DNP extracts. The RSC and the IC_50_ values for O_2_^•−^ were evaluated as mentioned above for the DPPH^•^ radical. Ascorbic acid was used as positive control. At least three independent experiments were performed for each tested sample.

### 4.10. Reducing Power (RP) Assay

Reducing power was determined spectrophotometrically as described previously [69]. RP_0.5AU_ value showing the extract concentration caused absorbance of 0.5 at 700 nm was calculated from the graph plotted absorbance against extract concentration. The tested concentrations in this assay were from 0.004 to 0.068 mg/mL for LP extract, from 0.25 to 8 mg/mL for WNP extract and from 0.125 to 4 mg/mL for DNP extract. Ascorbic acid was used as positive control. At least three independent experiments were performed for each tested compound.

### 4.11. Cell Culture Conditions

Human colon LS174 cancer cell line was obtained from ATCC company (Manassas, VA, USA). Human mesenchymal stem cells (MSC) were provided from Βiohellenika company (Thessaloniki, Greece). LS174 cancer cells were cultured in normal Dulbecco’s modified Eagle’s medium (DMEM, Gibko, UK), while MSC cells were cultured in Dulbecco’s modified Eagle’s medium high glucose with stable glutamine and sodium pyruvate 2 mM L-glutamine (DMEM, Gibko, UK). Both DMEMs contained 10% (*v*/*v*) fetal bovine serum, 100 units/mL of penicillin and 100 units/mL of streptomycin (Gibko, UK). Both cell types were cultured in plastic disposable tissue culture flasks at 37 °C in 5% CO_2_.

### 4.12. XTT Assay for Inhibition of Cell Proliferation

Cell proliferation inhibition was assessed using the XTT assay kit (Roche, Germany), as described previously [69]. Briefly, 1 × 10^4^ cells were subcultured into a 96-well plate in DMEM medium. After 24 h incubation, the cells were treated with different concentrations of each extract in serum-free DMEM medium for 24 h. Specifically, the concentrations were from 0.015 to 0.25 mg/mL for LP extract and from 0.25 to 4 mg/mL for WNP and DNP extracts in LS174 cells. In MSC cells, the concentrations were from 0.03 to 0.25 mg/mL for LP extract and from 0.25 to 4 mg/mL for WNP and DNP extracts in LS174 cells. Afterwards, 50 μL of XTT test solution, which was prepared by mixing 50 μL of XTT-labeling reagent with 1 μL of electron coupling reagent, was then added to each well. After 4 h of incubation, absorbance was measured at 450 nm and also at 690 nm as a reference wavelength on a Perkin Elmer EnSpire Model 2300 Multilabel microplate reader (Waltham, MA, USA). Cells cultured in DMEM serum-free medium were used as a negative control. Also, the absorbance of each extract concentration alone in DMEM serum-free medium and XTT test solution was tested at 450 nm. The absorbance values shown by the extracts alone were subtracted from those derived from cancer cell treatment with extracts. Data were calculated as percentage of inhibition by the following formula:

Inhibition (%) = [(O.D._control_ − O.D._sample_)/O.D._control_] × 100 (5)

where O.D.control and O.D.sample indicated the optical density of the negative control and the tested substances, respectively. The concentration of extract causing 50% cellular proliferation inhibition (IC_50_) of cancer cells was calculated thereafter from the graph plotted percentage inhibition against the extract concentration. All experiments were carried out on at least three separate occasions in triplicate.

### 4.13. Statistical Analysis

A non-parametric test was applied, as the primary analysis on both raw and log-transformed data indicated unequal variances. The Mann–Whitney U-test was used to determine significant differences in the investigated parameters and the Spearman rank coefficient to determine significant correlations between parameters. Differences were considered significant at *p* < 0.05.

## 5. Conclusions

The present study is the first that compared *P. oceanica* extracts from living leaves (i.e., LP), beach casts/‘*banquettes*’ (i.e., DNP) and dead leaves deposited on the coast at sea level where the waves break (i.e., WNP), regarding their polyphenolic content, antioxidant capacity and antiproliferative activity against cancer cells.

Thus, the present findings showed that high H_2_O_2_ level lad to oxidative stress in living leaves of *P. oceanica*. Then, the living leaves, as a response to oxidative stress, produced high amounts of phenolics, including chicoric acid, (-)-epigallocatechin gallate, *p*-coumaric acid, rutin hydrate, caftaric acid, *trans*-cinnamic, *trans*-ferulic acid and sinapinic acid, accounting for their extracts’ (i.e., LP) antioxidant and antiproliferative properties. Importantly, extracts of *P. oceanica’s ‘*Necromass’ (i.e., DNP and WNP extracts) retained, although at lower levels, the antioxidant and antiproliferative properties of LP extracts.

Given the *P. oceanica* meadows’ ecological value in the Mediterranean Sea, they are protected by international conventions. Our results suggested that if there is an acceptable management plan of *P. oceanica* meadows, which includes the collection of a sufficient amount of LP leaves, without adverse effects, *P. oceanica’s* living leaves could be exploited for developing high-added value products being beneficial for human health. In addition, it is a major problem that although *P. oceanica*’s beach casts/‘*banquettes*’ are also considered to be priority habitats, there is still no legislative framework for their management in Mediterranean countries, and they are often treated as waste and disposed in landfills. However, our results suggested that the Dry ‘Necromass’ material (DNP) could also be utilized for the production of high-added value products, provided that it would be followed by management programs (i.e., removal of up to 20% of their mass from the coasts).

## Figures and Tables

**Table 1:** Percentage (%) of the amount of each phenolic compound per total amount of phenols and per sample amount in living leaves (LP) and Dry ‘Necromass’ (DNP) of *P. oceanica*. Elution time for each phenolic compound.

Living Leaves (LP) (5000 mg L^−1^)				
Phenolic Compounds	Phenolic Compounds (mg)	Percentage (%) of Phenolic Compounds (mg)/Polyphenols (mg)	Percentage (%) of Phenolic Compounds (mg)/Sample (mg)	Elution Time (min)
Caftaric acid	0.014	1.176	0.162	4.66
Chicoric acid	0.625	51.229	7.059	12.50
Quercetin	-	-	-	-
Caffeic acid	-	-	-	-
(-)-Epigallocatechin gallate	0.348	28.542	3.933	10.46
*p*-Coumaric acid	0.170	13.903	1.916	11.87
*trans*-Ferulic acid	0.008 *	0.666 *	0.092 *	13.07
Sinapinic acid	0.017	1.371	0.189	13.59
Rutin hydrate	0.035	2.836	0.391	16.98
*trans*-Cinnamic acid	0.003 *	0.277 *	0.038 *	20.77
Hesperidin	-	-	-	-
4′,5,7-Trihydroxyflavone	-	-	-	-
**Total phenolic content (mg)**	1.221		13.781	
**Amount of samples (mg)**	8.86			
**Dry ‘Necromass’ (DNP) (7000 mg L^−1^)**				
Caftaric acid	-	-	-	-
Chicoric acid	-	-	-	-
Quercetin	-	-	-	-
Caffeic acid	T	T	T	9.55
(-)-Epigallocatechin gallate	0.010 *	59.377 *	0.097 *	10.44
*p*-Coumaric acid	-	-	-	-
*trans*-Ferulic acid	-	-	-	-
Sinapinic acid	-	-	-	-
Rutin hydrate	-	-	-	
*trans*-Cinnamic acid	0.003 *	15.335 *	0.025 *	20.78
Hesperidin	-	-	-	-
4′,5,7-Trihydroxyflavone	-	-	-	-
**Total phenolic content (mg)**	0.018		0.168	
**Amount of samples (mg)**	10.74			

T: Trace (i.e. amounts below the detection limit of the method). (*): values below the limit of quantification.

**Table 2:** Correlation (Spearman’s correlation coefficient, *p* values) between enzymes’ activity (i.e., APX, SOD), protein content and oxidative stress value (CTCF) in all the tested *Posidonia oceanica* samples, between the bioactive assays’ values (DPPH^•^, ABTS^•+^, OH^•^, O_2_^•−^, RP), and between the bioactive assays’ values and the phenolic content ^•+^ in all *P. oceanica* extracts.

	r	n
APX–SOD activity	0.895 ***	12
APX–protein content	−0.930 ***	12
SOD–protein content	−0.951 ***	12
APX–CTCF value	−0.710 *	12
SOD–CTCF value	−0.621 *	12
protein content–CTCF value	0.680 *	12
DPPH^•^–ABTS^•+^	0.783 *	9
DPPH^•^–OH^•^	0.943 **	6
DPPH^•^–O_2_^•−^	0.917 **	9
DPPH^•^–RP_0.5AU_	0.870 **	9
ABTS^•+^–OH^•^	0.600 ^ns^	6
ABTS^•+^–O_2_^•−^	0.850 **	9
ABTS^•+^–RP_0.5AU_	0.812 **	9
OH^•^–O_2_^•−^	0.657 ^ns^	6
OH^•^–RP_0.5AU_	0.638 ^ns^	6
O_2_^•−^–RP_0.5AU_	0.879 **	9
DPPH^•^–phenolic content	−0.867 **	9
ABTS^•+^–phenolic content	−0.600 ^ns^	9
OH^•^–phenolic content	−1.000 ***	6
O_2_^•c^–phenolic content	−0.683 *	9
RP–phenolic content	−0.812 **	9

* *p* < 0.05, ** *p* < 0.01, *** *p* < 0.001, ^ns^: not significant at 0.05 level, n: number of the measured values. r: correlation coefficient.

## Data Availability

The data presented in this study are available on request from the corresponding authors.

## Supplementary Materials

The following supporting information can be downloaded at https://www.mdpi.com/article/10.3390/md22030130/s1. Figure S1: scavenging activities against DPPH^•^, ABTS^•+^ and O_2_^•−^ radicals in the *Posidonia oceanica* living leaf (LP), wet necromass (WNP) and dry necromass (DNP) extracts. Figure S2: scavenging activities against OH^•−^ radical, and reducing power activity, in the *Posidonia oceanica* living leaf (LP), Wet Necromass (WNP) and Dry Necromass (DNP) extracts. Table S1: ascorbic acid’s IC_50_ values of scavenging activity against DPPH^•^, ABTS^•+^, OH^•^ and O2^•−^ radicals, as well as its RP_0.5AU_ value in RP assay. Table S2: synopsis of phenolic compounds detected in the *Posidonia oceanica* seagrass.
