# Supplementary material for: Antioxidant Activity, Inhibition of Intestinal Cancer Cell Growth and Polyphenolic Compounds of the Seagrass Posidonia oceanica’s Extracts from Living Plants and Beach Casts
Source: Mar Drugs. 2024 Mar 11;22(3):130. doi: 10.3390/md22030130 (PMC10972234; doi:10.3390/md22030130)
Supplement: Supplementary file 1 [file marinedrugs-22-00130-s001.zip › Table S2.pdf]

Table S2. Synopsis of phenolic compounds detected in the *Posidonia oceanica* seagrass.

**Phenolic compounds**	**References**
Acetosyringone	42,43
p-Anisic acid	42,43
p-OH Benzoic acid	42,43
Caftaric acid	98
Caffeic acid	42,42
Catechin	101
Chicoric acid	44,98,101
Chlorogenic acid	109
Cinnamic acid	42,43
p-Coumaric acid	42,43
Ferulic acid	42
Gallic acid	43,93
Gentisic acid	44
p-Hydroxybenzoic acid	42,43
4-O-p-Hyxdroxybenzoyl-vanillic acid, 3-Methoxy-4-hydroxy-benzoic acid	109
Phloridzin, Phloroglucinol, 4-Hydroxybenzoic acid	42
Piceol, Acetovanillone	43
Protocatechuic acid	43
Acetosyringone, Syringic acid, Acetovanillone, Pyrocatechol, Benzoic acid, p-Anisic acid, 4-OH benzoic acid, Phloroglucinol, Protocatechuic acid, Pyrogallol	93
Quercetin	17
Syringic acid	42,43
Sinapinic acid, Acetosyringone	42
Vanillin	43
Vanillic acid	42,43,109
