# Supplementary material for: Antioxidant Activity, Inhibition of Intestinal Cancer Cell Growth and Polyphenolic Compounds of the Seagrass Posidonia oceanica’s Extracts from Living Plants and Beach Casts
Source: Mar Drugs. 2024 Mar 11;22(3):130. doi: 10.3390/md22030130 (PMC10972234; doi:10.3390/md22030130)
Supplement: Supplementary file 1 [file marinedrugs-22-00130-s001.zip › Figure S1.pdf]

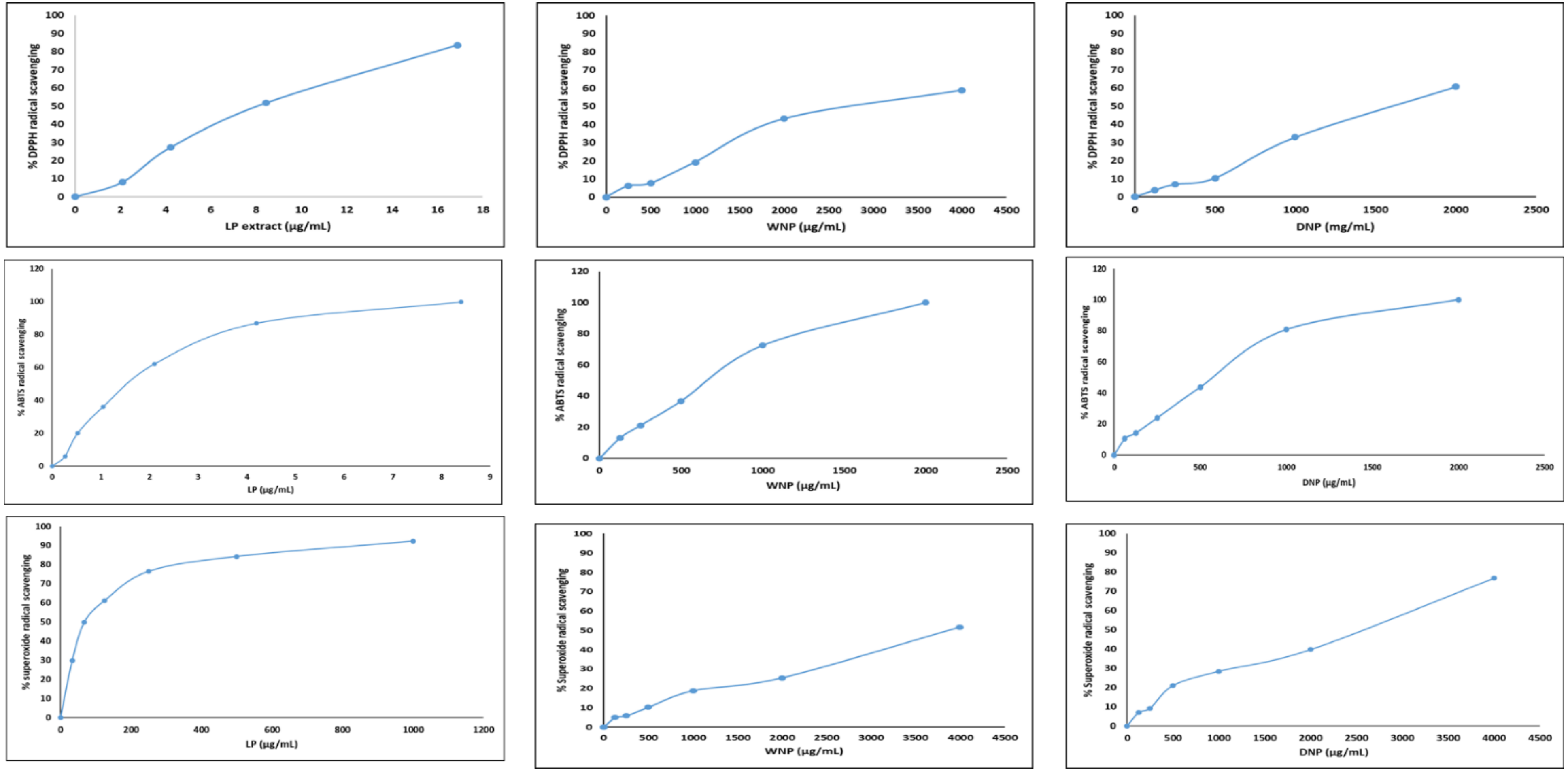

Figure S1. Scavenging activities against $DPPH^{\bullet}$, $ABTS^{\bullet +}$, and $O2^{\bullet -}$radicals in the *Posidonia oceanica* living leaf (LP), wet necromass (WNP) and dry necromass (DNP) extracts.
