# Supplementary material for: Antioxidant Activity, Inhibition of Intestinal Cancer Cell Growth and Polyphenolic Compounds of the Seagrass Posidonia oceanica’s Extracts from Living Plants and Beach Casts
Source: Mar Drugs. 2024 Mar 11;22(3):130. doi: 10.3390/md22030130 (PMC10972234; doi:10.3390/md22030130)
Supplement: Supplementary file 1 [file marinedrugs-22-00130-s001.zip › Table S1.pdf]

Table S1. Ascorbic acid's IC_{50} values of scavenging activity against $DPPH^{\bullet}$, $ABTS^{\bullet+}$, $OH^{\bullet}$, and $O_2^{\bullet-}$ radicals as well as its $RP_{0.5AU}$ value in RP assay.

	IC_{50} (mg/mL)				$RP_{0.5AU}$ (mg/mL)
	**$DPPH^{\bullet}$ [a]**	**$ABTS^{\bullet+}$ [a]**	**$^{\bullet}OH$ [a]**	**$O_2^{\bullet-}$ [a]**	**RP [a]**
Positive control					
Ascorbic acid	0.004 ± 0.0003 *	0.005 ± 0.0001 *	0.212 ± 0.009 *	ND	0.004 ± 0.0002 *

[a] Values are the mean ± SD of at least three separate triplicate experiments. ND: Not determined IC_{50} values (i.e. in the $O_2^{\bullet-}$ assay, ascorbic acid could not be tested because it can reduce NBT).
